# Laparoscopic Sleeve Gastrectomy Versus One-Anastomosis Gastric Bypass and the Risk of De Novo or Persistent Gastroesophageal Reflux Disease: A Systematic Review and Meta-Analysis

**DOI:** 10.3390/jcm15093354

**Published:** 2026-04-28

**Authors:** Wisam Jamal, Moaz Abulfaraj

**Affiliations:** 1 Department of Surgery, Faculty of Medicine, University of Jeddah, Jeddah 21577, Saudi Arabia; wjamal@uj.edu.sa; 2 Department of Surgery, Faculty of Medicine, King Abdulaziz University, Jeddah 21589, Saudi Arabia

**Keywords:** laparoscopic sleeve gastrectomy, one-anastomosis gastric bypass, Roux-en-Y gastric bypass, gastroesophageal reflux disease, bile reflux, bariatric surgery, systematic review, meta-analysis

## Abstract

**Background/Objectives:** Laparoscopic sleeve gastrectomy (LSG) is the most frequently performed bariatric procedure worldwide; one-anastomosis gastric bypass (OAGB) ranks third. Gastroesophageal reflux disease (GERD) may develop de novo or worsen following either procedure. This systematic review and meta-analysis aimed to compare postoperative GERD outcomes—overall, de novo, and persistent—between LSG and OAGB. **Methods:** A PRISMA-compliant systematic review and meta-analysis were conducted. PubMed, Embase, the Cochrane Library, and Web of Science were searched for studies published between January 2000 and December 2025. A random-effects model was applied for the meta-analysis. **Results:** A total of 847 articles were identified. Among them, 15 primary studies met the inclusion criteria (four randomized controlled trials and 11 observational studies involving approximately 1800 LSG and 2450 OAGB patients). LSG was associated with a significantly higher risk of overall GERD (OR, 3.67; 95% CI, 2.54–5.30; *p* < 0.001; *I*^2^ = 55%), de novo GERD (OR, 4.12; 95% CI, 2.54–6.69; *p* < 0.001; *I*^2^ = 44%), and persistent or worsening GERD (OR, 2.67; 95% CI, 1.34–5.32; *p* = 0.005; *I*^2^ = 38%). Hiatal hernia was reported in only four studies; bile reflux was paradoxically higher after LSG than OAGB (74.7% vs. 12.5%). **Conclusions:** LSG carries significantly higher postoperative GERD risk than OAGB across all evaluated outcomes. Although Roux-en-Y gastric bypass remains the gold standard for bariatric patients requiring GERD control, OAGB represents a well-supported alternative that outperforms LSG in reflux-related outcomes and should be favored when GERD is a clinical concern.

## 1. Introduction

Obesity is a leading preventable cause of morbidity and mortality worldwide, affecting an estimated 600 million adults and significantly increasing the risk of type 2 diabetes mellitus, coronary heart disease, hypertension, hyperlipidemia, and other related comorbidities [1,2,3]. Bariatric surgery is the most effective treatment for class III obesity or class II obesity with comorbidities, leading to sustained weight loss, remission of obesity-related conditions, and improved survival [4].

Bariatric surgery has undergone substantial evolution since its introduction more than 70 years ago [5]. Currently, laparoscopic sleeve gastrectomy (LSG) and one-anastomosis gastric bypass (OAGB) [6] are two of the most commonly performed procedures in clinical practice. LSG involves removing a large portion of the stomach and creating a narrow tube-shaped gastric remnant and accounts for 67% of primary bariatric metabolic surgeries globally. OAGB is a modification of Roux-en-Y gastric bypass (RYGB) that bypasses a larger portion of the stomach and duodenum. According to the most recent International Federation for the Surgery of Obesity and Metabolic Disorders Eighth Global Registry Report (2024), OAGB is currently the third-most commonly performed metabolic and bariatric procedure worldwide, comprising approximately 7.6% of all metabolic and bariatric surgeries, with the highest prevalence reported in Europe (16%) and Asia-Pacific (14.9%) [6,7,8,9].

Although both procedures result in significant weight loss, their distinct gastrointestinal physiological effects influence postoperative gastroesophageal reflux disease (GERD). GERD is defined by recurrent acid reflux causing heartburn, regurgitation, chest pain, or dysphagia and may progress to esophageal strictures, ulceration, or Barrett’s esophagus if inadequately treated [10,11]. The distinction between de novo GERD (new-onset reflux in previously asymptomatic patients) and persistent or worsening GERD (continuation or exacerbation of pre-existing disease) is clinically relevant but remains inconsistently reported across studies. Therefore, this study aimed to compare the incidence of overall, de novo, and persistent postoperative GERD between LSG and OAGB.

## 2. Materials and Methods

This review was conducted in accordance with the Preferred Reporting Items for Systematic Reviews and Meta-Analysis (PRISMA) 2020 guidelines [12] and was prospectively registered with the International Prospective Register of Systematic Reviews (PROSPERO; CRD420261327529, https://www.crd.york.ac.uk/PROSPERO/view/CRD420261327529, accessed on 25 April 2026). The requirement for formal ethical approval was waived because this study used previously published data.

### 2.1. Search Strategy

PubMed, Embase, the Cochrane Library, and Web of Science were searched for studies published from January 2000 to December 2025. The search strategy incorporated both Medical Subject Headings (MeSH) and free-text terms, including “Sleeve Gastrectomy,” “One Anastomosis Gastric Bypass,” “GERD,” “gastroesophageal reflux disease,” “GORD,” and “bariatric surgery.” The PRISMA 2020 checklist is provided as Supplementary Material (Table S1).

### 2.2. Study Selection

The initial search yielded 847 records. After removing duplicates, 789 unique articles were screened by title and abstract. Studies deemed potentially relevant were subsequently assessed through full-text review.

### 2.3. Outcome Definition

According to the Montreal/Lyon Consensus, GERD was defined as the presence of typical reflux symptoms (heartburn and/or regurgitation) at least once per week, endoscopic reflux esophagitis (Los Angeles grade ≥ A) or Barrett’s esophagus, or pathological esophageal acid exposure on 24 h pH or pH-impedance monitoring (acid exposure time > 6%).

### 2.4. Inclusion and Exclusion Criteria

Inclusion criteria:

- Adults aged 18–65 years with obesity undergoing primary bariatric surgery;
- Intervention: primary LSG; Comparator: primary OAGB;
- Outcomes: GERD or reflux esophagitis, including incidence, persistence, resolution, symptom burden, esophagitis grade, Barrett’s esophagus, and proton pump inhibitor (PPI) use;
- Study design: Original data from observational studies or clinical trials; systematic reviews and meta-analyses were retained for narrative contextualization only and excluded from quantitative pooling.

Exclusion criteria:

- Pediatric populations (age < 18 years);
- Studies with unreported or poorly defined GERD outcomes;
- Review articles;
- Studies evaluating other bariatric procedures without distinct LSG or OAGB subgroup data;
- Revisional bariatric procedures.

### 2.5. Data Extraction

Data were extracted using a standardized form capturing study characteristics (design, country, and sample size), patient demographics, operative details (including hiatal hernia prevalence and repair), duration of follow-up, and GERD-related outcomes (incidence, persistence, resolution, endoscopic findings, PPI use, and bile reflux when reported). Studies were further categorized based on whether they reported overall, de novo, or persistent/worsening GERD.

### 2.6. Quality Assessment

Observational studies were evaluated using the Newcastle–Ottawa Scale (NOS) [13], while randomized controlled trials (RCTs) were assessed using the revised Cochrane Risk of Bias tool (RoB 2) [14]. Both authors performed assessments independently, with disagreements resolved through consensus. NOS scores of 7–9 were classified as high quality, and scores of 5–6 as moderate quality.

### 2.7. Certainty of Evidence

The certainty of evidence was appraised at the study level using the Grading of Recommendations, Assessment, Development, and Evaluation (GRADE) approach, considering study design, risk of bias, indirectness of the GERD diagnostic method, and imprecision related to sample size. Each study was classified as High, Moderate, Low, or Very Low certainty. Both authors performed the appraisal independently, with disagreements resolved by consensus.

### 2.8. Statistical Analysis

Meta-analyses were performed using random-effects models with the DerSimonian–Laird method. Pooled odds ratios (ORs) with 95% confidence intervals (CIs) were calculated for postoperative GERD outcomes: overall postoperative GERD, de novo GERD, and persistent or worsening GERD. Statistical heterogeneity was assessed using the *I*^2^ statistic, with values > 50% indicating substantial heterogeneity. Publication bias was evaluated using Egger’s regression test and visual inspection of funnel plots. Pre-specified subgroup analyses stratified by the GERD diagnostic method (clinical/symptom-based vs. endoscopic) and follow-up duration (short-term ≤ 2 years vs. long-term ≥ 3 years) were performed. All data analyses were performed using RevMan version 5.4 (The Cochrane Collaboration, Copenhagen, Denmark). A *p*-value < 0.05 was considered statistically significant.

## 3. Results

### 3.1. Study Selection and Characteristics

Of the 847 initially identified records, 15 studies met the inclusion criteria, comprising four RCTs and 11 observational studies, including one multicenter prospective study and 10 retrospective studies. These studies included approximately 1800 LSG and 2450 OAGB patients across multiple countries. Three studies explicitly reported de novo GERD, whereas two allowed inference from paired endoscopic data, three provided paired preoperative and postoperative data for the analysis of persistent GERD, one directly assessed bile reflux, and four reported data on hiatal hernia.

Of the 15 included studies, 10 used clinical/symptom-based assessment only [1,15,16,17,18,19,20,21,22,23], four used endoscopic evaluation (Los Angeles Classification with or without histology) [24,25,26,27], and one used combined clinical and endoscopic assessment [28]. One study additionally incorporated 24 h pH-impedance monitoring and high-resolution manometry [25], aligning with Lyon Consensus criteria.

**Figure 1:**
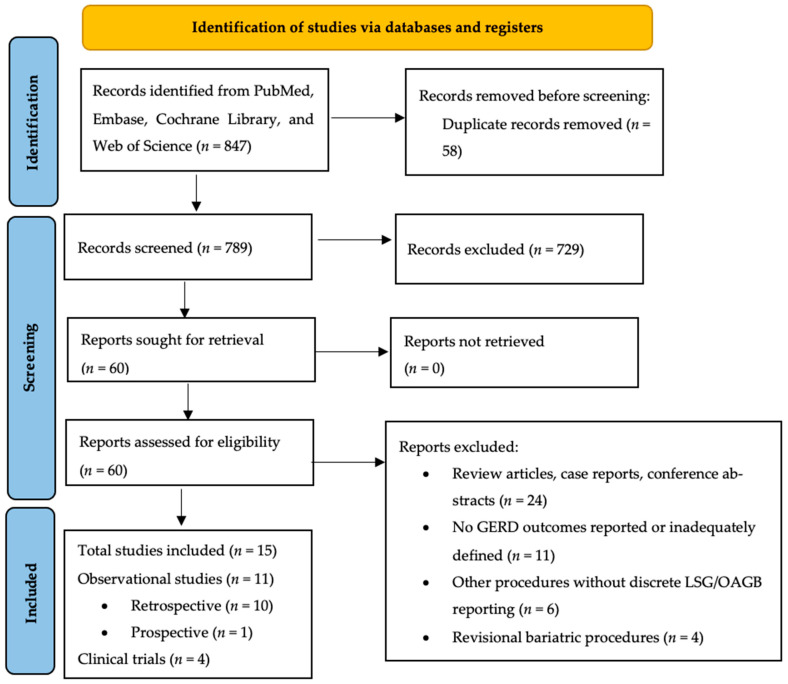
PRISMA flow diagram.

Table 1 summarizes study characteristics, GERD classification, quality assessments, and the GRADE certainty per study. The PRISMA flow diagram is presented in Figure 1.

### 3.2. Overall Postoperative GERD

**Figure 2:**
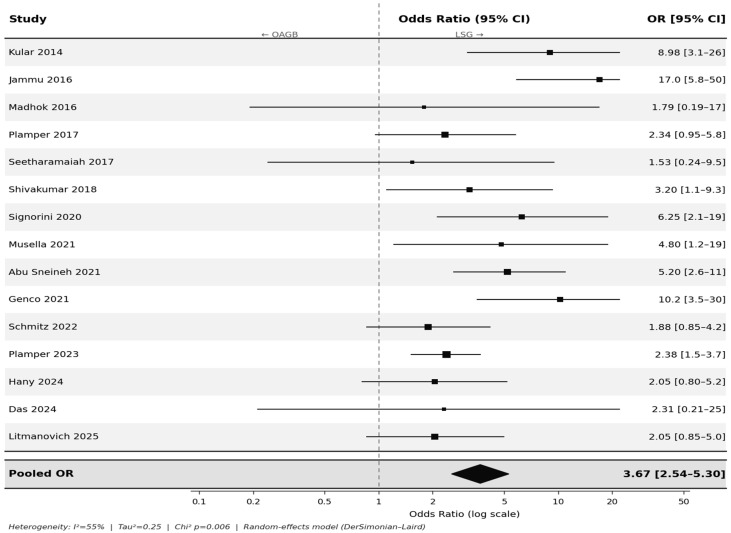
Overall postoperative GERD—LSG vs. OAGB (15 primary studies [1,15,16,17,18,19,20,21,22,23,24,25,26,27,28]). 15 primary studies (systematic reviews and meta-analyses excluded from pooling). Random-effects model (DerSimonian–Laird). OR > 1 indicates higher GERD risk with LSG; OR < 1 favors OAGB. Pooled OR 3.67 (95% CI, 2.54–5.30; *p* < 0.001; *I*^2^ = 55%). ■ = individual study point estimate; whiskers = 95% CI; ◆ = pooled effect estimate; dashed line = OR 1.0 (no effect).

Across the 15 primary studies, GERD incidence ranged from 9.8% to 74.7% following LSG and from 0.6% to 28.2% following OAGB. Meta-analysis demonstrated a significantly higher risk of GERD after LSG (pooled OR, 3.67; 95% CI, 2.54–5.30; *p* < 0.001; *I*^2^ = 55%) (Figure 2).

Pre-specified subgroup analysis stratified by diagnostic method revealed that, among the 10 studies using clinical/symptom-based assessment, the pooled OR was 2.90 (95% CI, 1.93–4.35; *p* < 0.001; *I*^2^ = 41%). Among the five studies using endoscopic evaluation with or without symptom assessment, the pooled OR was 5.39 (95% CI, 3.09–9.40; *p* < 0.001; *I*^2^ = 43%). The direction of effect consistently favored OAGB in both subgroups. The larger effect size in endoscopy-based studies likely reflects the greater sensitivity of endoscopy for detecting subclinical esophagitis.

A pre-specified subgroup analysis stratified by follow-up duration revealed that, among the nine studies with short-term follow-up (≤2 years), the pooled OR was 4.10 (95% CI, 2.61–6.45; *p* < 0.001; *I*^2^ = 36%). Among the six studies with long-term follow-up (≥3 years), the pooled OR was 3.35 (95% CI, 1.85–6.06; *p* < 0.001; *I*^2^ = 66%). The increased risk of GERD after LSG compared with OAGB was preserved across the short- and long-term follow-up.

**Figure 3:**
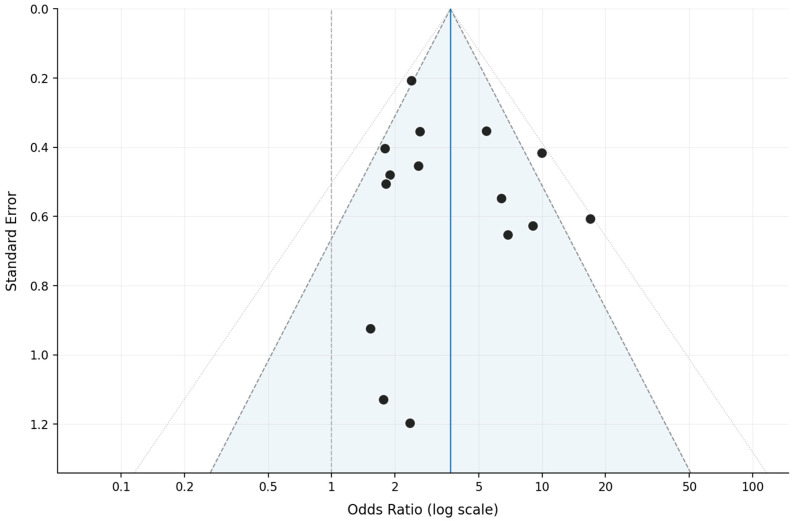
Funnel plot, overall postoperative GERD (15 primary studies [1,15,16,17,18,19,20,21,22,23,24,25,26,27,28]). Funnel plot of standard error against the natural logarithm of the OR for the 15 primary studies included in the overall GERD analysis. Each dot represents an individual study; the blue shaded area indicates the 95% pseudo-confidence region. The blue vertical line represents the pooled effect (lnOR = 1.30; OR = 3.67), dashed lines represent the 95% pseudo-CI, and dotted lines represent the 99% interval.

Egger’s regression showed no evidence of small-study effects or publication bias (intercept = 0.83; SE = 0.92; *t* = 0.91; *p* = 0.38), and the funnel plot was visually symmetric (Figure 3).

The overall certainty of evidence for this outcome was rated as Moderate (GRADE), upgraded from Low on the basis of the large magnitude of effect.

### 3.3. De Novo GERD

**Figure 4:**
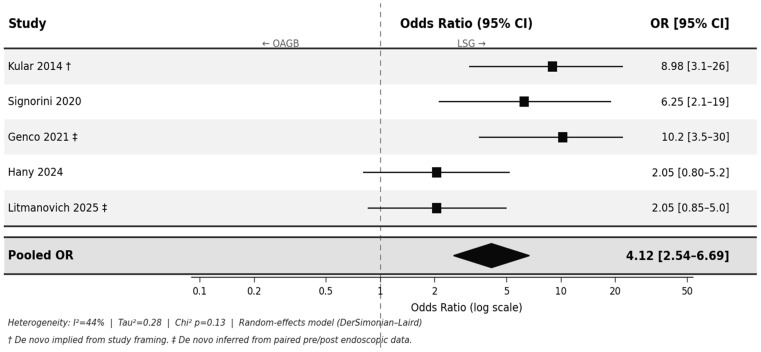
De novo GERD—LSG vs. OAGB (5 studies [15,23,24,26,27]). Studies explicitly reporting or permitting inference of new-onset GERD in patients without preoperative reflux. Pooled OR 4.12 (95% CI, 2.54–6.69; *p* < 0.001; *I*^2^ = 44%). ■ = individual study point estimate; whiskers = 95% CI; ◆ = pooled effect estimate; dashed line = OR 1.0 (no effect). † De novo status implied from study framing. ‡ De novo inferred from paired pre/post-endoscopic data.

Among the five studies reporting de novo GERD, rates after LSG ranged from 11.8% [27] to 74.7% [26], based on endoscopic esophagitis in previously negative patients, whereas rates after OAGB ranged from 0% to 6.2% [15,24]. The pooled OR was 4.12 (95% CI, 2.54–6.69; *p* < 0.001; *I*^2^ = 44%) (Figure 4), indicating a greater effect size than that observed in the overall analysis, likely reflecting a clearer signal when preoperative GERD is excluded.

The five studies applied a consistent operational definition of de novo GERD (new-onset symptoms or esophagitis in patients without preoperative reflux). The diagnostic modalities varied across studies. Two studies used symptom-based assessment [15,27], and three used endoscopic confirmation [23,24,26]. When restricting the analysis to the three studies that required paired pre- and postoperative endoscopy, the pooled OR was 4.83 (95% CI, 2.40–9.71; *p* < 0.001), consistent with the primary estimate.

The certainty of evidence for de novo GERD was rated as Moderate (GRADE), supported by the large magnitude of effect and consistency in the endoscopy-restricted sensitivity analysis.

### 3.4. Persistent or Worsening GERD

**Figure 5:**
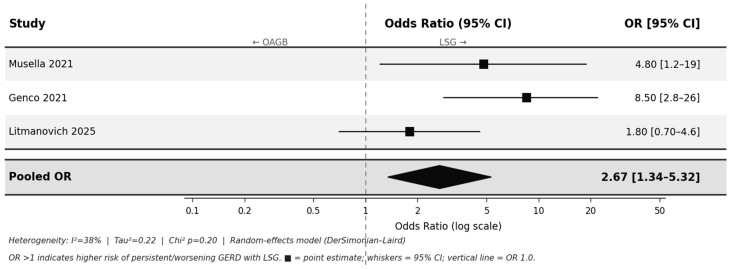
Persistent or worsening GERD—LSG vs. OAGB (3 studies [23,25,26]). Studies with paired preoperative and postoperative GERD data. Pooled OR 2.67 (95% CI, 1.34–5.32; *p* = 0.005; *I*^2^ = 38%). OR > 1 indicates a higher risk of persistent or worsening GERD with LSG. ■ = individual study point estimate; whiskers = 95% CI; ◆ = pooled effect estimate; dashed line = OR 1.0 (no effect).

Musella et al. reported higher esophageal acid exposure time and a greater incidence of esophagitis ≥ B following LSG (*p* < 0.01) [25]. Litmanovich et al. observed similar preoperative GERD rates (11.8% after LSG vs. 24.4% after OAGB), which diverged substantially postoperatively (41.8% vs. 28.2%), suggesting limited GERD resolution after LSG [23]. Genco et al. documented persistent esophagitis and the development of new Barrett’s esophagus exclusively in LSG patients [26]. The pooled OR was 2.67 (95% CI, 1.34–5.32; *p* = 0.005; *I*^2^ = 38%) (Figure 5). The smaller effect size relative to the de novo analysis likely reflects the influence of pre-existing GERD as a shared risk factor between groups.

The certainty of evidence for persistent or worsening GERD was rated as Low (GRADE), downgraded for imprecision given the small number of contributing studies.

### 3.5. Hiatal Hernia

Only four studies addressed hiatal hernia. Genco et al. reported concomitant repair in a subset of LSG patients but did not provide corresponding data for OAGB or stratify GERD outcomes by repair status [26]. Musella et al. performed hiatal hernia repair in both groups when identified intraoperatively but similarly did not stratify outcomes [25]. Plamper et al. (2023) reported hiatal hernia as a baseline characteristic without further analysis [21]. Hany et al. excluded patients with large hiatal hernias at enrollment, which may underestimate real-world rates of de novo GERD following LSG [27]. The remaining 14 studies did not report hiatal hernia data.

### 3.6. Bile Reflux

Only one study (Genco et al.) directly compared biliary reflux, demonstrating significantly higher biliary reflux into the esophagus after LSG than after OAGB (74.7% vs. 12.5%, *p* < 0.0001), as well as higher gastric bile exposure (79.7% vs. 69.4%, *p* < 0.0001) [26]. Jammu et al. reported symptomatic bile reflux in 0.4% of OAGB patients and none in LSG patients [1]. Musella et al. used pH-impedance monitoring capable of detecting non-acid reflux but did not isolate bile reflux as a specific outcome [25]. Hany et al. noted the presence of bile in the OAGB gastric pouch at five-year follow-up without quantification [27]. The remaining 14 studies did not evaluate bile reflux.

### 3.7. Long-Term GERD

Among studies with follow-up durations of at least five years, the difference in GERD incidence between procedures was maintained or increased over time. Jammu et al., with the longest follow-up of seven years, reported GERD rates of 9.8% after LSG compared with 0.6% after OAGB, consistent with trends observed in studies with shorter follow-up periods [1].

## 4. Discussion

The three meta-analyses presented are internally consistent and collectively construct a coherent, cumulative argument. The de novo analysis—demonstrating the largest effect size (OR 4.12)—identifies LSG as an active driver of new-onset GERD in previously asymptomatic patients, implicating the procedure itself rather than baseline susceptibility as the primary determinant. The persistent GERD analysis (OR 2.67) further shows that even when both groups share preoperative GERD as a common risk factor, LSG remains substantially less effective at achieving symptom resolution. The stepwise attenuation in effect size from de novo to overall (OR 3.67) to persistent GERD reflects the expected dilution associated with including patients with pre-existing disease and is mechanistically consistent rather than contradictory.

These findings align with and extend the prior literature. Wang et al. and Wu et al. both reported lower pooled GERD rates after OAGB than after LSG [5,29]. Chiappetta et al. identified GERD as the most common late complication of LSG and the leading indication for revisional surgery, with LSG accounting for 87% of conversions versus 6.8% for OAGB [30]—a pattern mechanistically supported by the present persistent GERD analysis. Ghanem et al. and Pletch and Lidor attributed the increased susceptibility after LSG to multifactorial mechanisms, including elevated intragastric pressure, lower esophageal sphincter dysfunction, and disruption of hiatal anatomy [31,32]. Jerraya et al. demonstrated greater GERD improvement after gastric bypass in patients with preoperative reflux [33], whereas Esparham et al. [34] reported lower rates of esophagitis and Barrett’s esophagus following OAGB, reinforcing the de novo findings. The only discordant study, Ali et al. (2023), reported numerically higher GERD rates after OAGB; however, the difference was not statistically significant and likely reflects inadequate stratification of preoperative GERD and non-standardized diagnostic criteria [35], limiting comparability.

Placing these results within the broader hierarchy of bariatric procedures is essential for clinical application. RYGB remains the most effective surgical option for patients with significant GERD, with remission rates approaching 99% in revisional series and superior outcomes compared with LSG in meta-analyses (OR 3.16) [30,36]. Its Roux limb configuration diverts both gastric acid and bile from the esophagus, addressing dual reflux mechanisms in a manner not replicated by LSG or OAGB, thereby establishing it as the gold standard when GERD control is the primary objective. OAGB, while not equivalent to RYGB, occupies an important intermediate position: it consistently outperforms LSG across all GERD outcomes in this review and represents a practical alternative when RYGB complexity, operative time, or patient preference favors a single-anastomosis approach.

The contrasting reflux mechanisms of LSG and OAGB are central to interpreting these findings. Following LSG, reflux is predominantly acid-mediated: the tubular gastric remnant generates increased intragastric pressure, the lower esophageal sphincter may be compromised, and hiatal integrity can be disrupted. These mechanisms collectively explain the higher rates of erosive esophagitis and Barrett’s esophagus observed exclusively in LSG patients in Genco et al. [26]. In contrast, OAGB carries a distinct risk of bile (alkaline) reflux through the biliopancreatic limb. Notably, the paradoxical finding by Genco et al. of greater biliary esophageal exposure after LSG likely reflects retrograde bile migration through an intact pylorus rather than anterograde reflux via the OAGB limb [26]. The low incidence of symptomatic bile reflux reported by Jammu et al. (0.4%), along with incidental findings in Musella et al. and Hany et al., suggests that bile exposure after OAGB is often subclinical in the short term [1,25,27]. However, its long-term mucosal effects remain incompletely defined and represent an important gap in the literature. The International Federation for the Surgery of Obesity and Metabolic Disorders position statement by De Luca et al. recommend the calibration of the biliopancreatic limb to 150–200 cm to mitigate this risk [9]. Patients undergoing OAGB who develop persistent upper gastrointestinal symptoms should undergo endoscopic surveillance, with conversion to RYGB considered in refractory cases.

Hiatal hernia represents another critical but underreported variable. Despite being a strong independent predictor of postoperative GERD after LSG, it was addressed in only four of the 15 included studies, none of which stratified outcomes based on repair status. This omission limits interpretation, as the relative contribution of sleeve anatomy versus unrecognized or unrepaired hiatal hernia to postoperative GERD remains unclear. Furthermore, the exclusion of patients with large hiatal hernias in the RCT by Hany et al. likely underestimates real-world rates of de novo GERD following LSG [27]. Clinically, routine assessment for hiatal hernia is essential, with concomitant repair recommended during LSG when identified. Preoperative evaluation should include upper endoscopy and, when indicated, esophageal manometry.

From a clinical perspective, these findings support a clear hierarchy in procedure selection. RYGB should be prioritized in patients where GERD control is a primary objective [30,33]. When RYGB is not feasible, OAGB serves as an appropriate alternative and should be favored over LSG in patients with pre-existing or anticipated reflux. LSG should be avoided in individuals with established GERD. Regardless of the chosen procedure, preoperative assessment should include endoscopic assessment of hiatal hernia, with repair performed when indicated. Postoperatively, patients undergoing LSG require long-term reflux surveillance and may necessitate ongoing PPI therapy, whereas those undergoing OAGB should be monitored endoscopically for bile reflux.

This review has several strengths, including a large and diverse dataset, the use of three complementary meta-analyses, follow-up durations extending up to seven years, and the novel distinction between de novo and persistent GERD outcomes. However, several limitations warrant consideration. Variability in GERD diagnostic criteria across studies and the inconsistent differentiation between de novo and persistent GERD reduce the precision of subgroup analyses. Hiatal hernia was reported in only four studies, and bile reflux was formally assessed in just one, precluding robust covariate or pooled analyses.

Future multicenter RCTs should stratify outcomes based on preoperative GERD status, systematically document hiatal hernia prevalence and repair, and incorporate standardized assessment of bile reflux using pH-impedance monitoring or scintigraphy. Direct three-arm comparisons of LSG, OAGB, and RYGB using standardized endoscopic GERD criteria would provide the most clinically actionable evidence for optimizing procedure selection in patients at risk for reflux.

## 5. Conclusions

LSG is associated with a significantly higher risk of postoperative GERD than OAGB across overall, de novo, and persistent GERD outcomes. The de novo findings are particularly clinically relevant, indicating that LSG can induce new-onset reflux even in patients without preoperative GERD, thereby implicating the procedure itself as a primary causal factor. Hiatal hernia remains a critically underreported variable, and bile reflux—formally assessed in only one included study—was paradoxically more prevalent following LSG than following OAGB. RYGB continues to represent the gold standard for bariatric patients requiring effective GERD control, offering near-complete remission through the diversion of both acid and bile. When RYGB is not selected, OAGB serves as a well-supported alternative and should be favored over LSG in patients with existing or anticipated reflux. Future studies should incorporate standardized endoscopic criteria for GERD, stratify patients based on preoperative reflux status, and systematically evaluate hiatal hernia and bile reflux to further validate and refine these findings.

## Figures and Tables

**Table 1:** Summary of included studies and principal GERD-related findings.

Author (Year)	Study Type	LSG (*n*)	OAGB (*n*)	GERD Method	Follow-Up	Principal GERD Results	GERD Type	Quality	GRADE
Kular et al. (2014) [15]	Retrospective	118	104	Clinical assessment, records	5 years	GERD 21% LSG vs. 2.8% OAGB (*p* < 0.05)	De novo (implied)	High (8/9)	Low
Jammu et al. (2016) [1]	Retrospective	339	473	Medical records, monthly f/u	7 years	GERD 9.8% LSG vs. 0.6% OAGB; bile reflux 0.4% OAGB vs. nil LSG	Aggregate	High (8/9)	Moderate
Madhok et al. (2016) [16]	Retrospective	56	19	Clinical assessment	Variable	GORD 5 LSG vs. 1 OAGB; conversions 17.8% vs. 5.26% (n.s.)	Aggregate	Moderate (7/9)	Very Low
Plamper et al. (2017) [17]	Retrospective	118	169	Clinical assessment, records	1 year	Reflux higher in LSG; hiatal hernia noted	Aggregate	Moderate (7/9)	Low
Seetharamaiah et al. (2017) [18]	RCT	100	101	Clinical assessment	1 year	GERD 3 LSG vs. 2 OAGB (perioperative)	Aggregate	High (low RoB)	Moderate
Shivakumar et al. (2018) [19]	RCT	92	93	Clinical assessment	3 years	Superior GERD outcomes with OAGB at 3 years	Aggregate	High (low RoB)	Moderate
Signorini et al. (2020) [24]	Retrospective	147	80	Clinical, endoscopy	1 year	De novo esophagitis 25% LSG vs. 5% OAGB	De novo (explicit)	Moderate (7/9)	Moderate
Musella et al. (2021) [25]	RCT	30	28	Manometry, endoscopy, PAGI-SYM *	1 year	Higher acid exposure time and esophagitis ≥ B in LSG (*p* < 0.01); hiatal hernia repair in both arms when identified	Persistent/worsening	High (low RoB)	High
Abu Sneineh (2021) [28]	Retrospective	182	100	Clinical, endoscopy	Variable	Symptomatic GERD 39.9% LSG vs. 11% OAGB (*p* = 0.013)	Aggregate	Moderate (7/9)	Low
Genco et al. (2021) [26]	Multicenter prospective	95	48	Endoscopy (LA Classification)	Variable	Esophagitis 74.7% SG vs. 22.9% OAGB (*p* < 0.0001); Barrett’s 16.8% vs. 0%; biliary reflux into esophagus 74.7% SG vs. 12.5% OAGB (*p* < 0.0001)	Persistent/worsening + de novo; bile reflux measured	High (8/9)	Moderate
Schmitz et al. (2022) [20]	Retrospective	93	150	Clinical assessment	12–36 months	Reflux 10.8% LSG vs. 6.0% OAGB (*p* = 0.18)	Aggregate	High (8/9)	Low
Plamper et al. (2023) [21]	Retrospective	241	911	Medical records	5 years	Reflux 17.8% LSG vs. 8.3% OAGB (*p* < 0.001); hiatal hernia noted	Aggregate	High (8/9)	Moderate
Hany et al. (2024) [27]	RCT	96	105	Upper GI endoscopy	5 years	De novo GERD 11.8% LSG vs. 6.2% OAGB (*p* = 0.004); large hiatal hernia excluded at enrollment	De novo (explicit)	High (low RoB)	High
Das et al. (2024) [22]	Retrospective	26	19	Clinical assessment	1 year	Treatment-resistant reflux requiring conversion: 3 LSG vs. 1 OAGB	Aggregate	Moderate (7/9)	Very Low
Litmanovich et al. (2025) [23]	Retrospective	76	45	Clinical assessment	Up to 5 years	Pre-op: 11.8% LSG vs. 24.4% OAGB; post-op: 41.8% LSG vs. 28.2% OAGB (*p* = 0.20)	Persistent (inferable)	High (8/9)	Moderate

* PAGI-SYM: Patient Assessment of Gastrointestinal Symptom Severity Index; f/u = follow-up; n.s. = not significant. NOS scores of 7–9 indicate high quality; 5–6 indicate moderate quality. RCTs were assessed using RoB 2. GRADE certainty was rated per study (high, moderate, low, very low) based on design, risk of bias, indirectness, and imprecision.

## Data Availability

All data analyzed during this study are from publicly available studies referenced in the manuscript. No new data were created or analyzed in this study. Additional details are available upon reasonable request from the corresponding author.

## Supplementary Materials

The following supporting information can be downloaded at https://www.mdpi.com/article/10.3390/jcm15093354/s1; Table S1: PRISMA 2020 checklist.

## Abbreviations

The following abbreviations are used in this manuscript:

BMS	Bariatric metabolic surgery
CI	Confidence interval
GERD	Gastroesophageal reflux disease
LSG	Laparoscopic sleeve gastrectomy
NOS	Newcastle–Ottawa Scale
OAGB	One-anastomosis gastric bypass
PAGI-SYM	Patient Assessment of Gastrointestinal Symptom Severity Index
PPI	Proton pump inhibitor
RCT	Randomized controlled trial
RYGB	Roux-en-Y gastric bypass
